# The complete mitochondrial genome of *aglaeactis castelnaudii* (bourcier & mulsant, 1848) (apodiformes: trochilidae: *aglaeactis*) and phylogenetic analysis

**DOI:** 10.1080/23802359.2024.2397980

**Published:** 2024-09-04

**Authors:** Guangshuai Liu, Jincheng Liu, Xinyue Zhang, Xiaodong Gao

**Affiliations:** College of Life Science, Qufu Normal University, Qufu, PR China

**Keywords:** *Aglaeactis castelnaudii*, mitochondrial genome, phylogenetic analysis

## Abstract

In this study, we employed high-throughput sequencing data to assemble the mitochondrial genome (mitogenome) of the White-tufted Sunbeam (*Aglaeactis castelnaudii*). The total length of the mitogenome was found to be 16,872 base pairs (bp), containing 13 protein-coding genes (PCGs), 22 transfer RNA genes, 2 ribosomal RNA genes, and 1 control region. The nucleotide composition was as follows: A 30.6%, T 24.0%, C 31.2%, and G 14.2%, resulting in a GC content of 45.4%. Phylogenetic analysis, utilizing the concatenation of the 13 mitochondrial PCGs, indicated a closer evolutionary relationship between the genus *Aglaeactis* and the genus *Coeligena* compared to other genera within the family Trochilidae investigated in this study. The mitogenome of *A. castelnaudii* not only contributes to species identification but also provides valuable insights for phylogenetic and conservation genetic analyses of *A. castelnaudii*.

## Introduction

**Figure 1.**
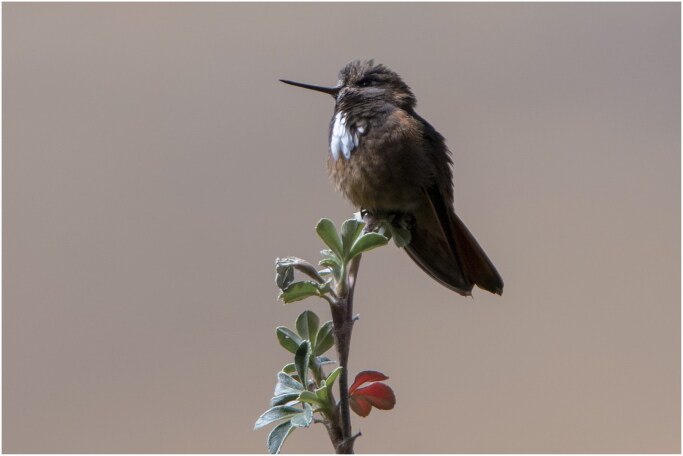
Image of an adult individual of *aglaeactis castelnaudii* (photo credit: David F. Belmonte, email: david.rguezpascual@gmail.com, used with permission). This photograph was captured in october 2022 in grau apurímac, Peru. The original photograph is accessible to the public on iNaturalist (https://www.inaturalist.org/photos/250606396?size=original).

The White-tufted Sunbeam, scientifically known as *Aglaeactis castelnaudii* (Figure 1), belongs to the hummingbird family Trochilidae. *A. castelnaudii* inhabits a restricted and fragmented range, exclusively found in the Andes of Peru, Bolivia, and Northern Chile. It prefers damp montane forests and high-altitude shrublands in subtropical or tropical regions (Parker & O'Neill 1980). Playing a significant role in ecosystem regulation, *A. castelnaudii* primarily feeds on insects or flower nectar from blooming plants in its Andean habitat (Watts 2008). The International Union for Conservation of Nature (IUCN) describes this species as 'common but patchily distributed’ within its habitat, although an accurate estimation of the overall population is currently lacking. As of 2017, the White-tufted Sunbeam is classified as ‘Near Threatened’ (NT) on the IUCN Red List, with its population size and trend assessed as ‘Decreasing’ (BirdLife International 2017).

The genus *Aglaeactis* comprises four species (Schuchmann 1985) and belongs to the Andean hummingbird clade (McGuire et al. 2014). All *Aglaeactis* hummingbirds inhabit the high Andes at elevations ranging from 2500 to 4300 m, where they have evolved several distinct adaptive phenotypes to survive in this challenging environment (Schuchmann 1990; Ocampo et al. 2020). Consequently, this group stands as a notable example of adaptive radiation in temperate and high latitudes. Remarkably, the genus *Aglaeactis* remains among the least researched hummingbird genera globally, with the mitogenome of this genus yet to undergo sequencing. In this study, we present and elucidate the mitogenome of *A. castelnaudii* for the first time. This study offers genetic data crucial for future studies into species identification, biodiversity surveillance, conservation genetics, and investigations into adaptive evolution.

## Materials and methods

In this study, the mitogenome of *A. castelnaudii* was assembled utilizing publicly available high-throughput genomic data in NCBI, which was submitted by the Sam Noble Oklahoma Museum of Natural History. An individual muscle tissue sample from *A. castelnaudii* was collected in Peru, situated at coordinates 14.058 S latitude and 73.001 W longitude. This sample was preserved at the Sam Noble Oklahoma Museum of Natural History (https://samnoblemuseum.ou.edu/, contact person: Jessica McLaughlin, email: jfmclaughlin@berkeley.edu) under the sample label ca169414. Genomic DNA was isolated from the muscle tissue and subjected to sequencing on an Illumina NovaSeq 6000 platform utilizing 150 bp paired-end runs (2 × 150). The genomic data supporting the findings of this study can be accessed at NCBI's Sequence Read Archive (SRA) *via* the following link: https://www.ncbi.nlm.nih.gov/sra/, with the accession number SRR19461672. Data collection was conducted in compliance with the IUCN policies research involving species at risk of extinction (see Guidelines for appropriate uses of IUCN Red list data). Furthermore, we adhered to the regulations of Convention on Biological Diversity and the Convention on the Trade in Endangered Species of Wild Fauna and Flora.

We extracted the mitochondrial reads from the genomic data and proceeded to perform assembly using MitoZ v3.6 (Meng et al. 2019). After a manual examination, the resulting mitogenome was annotated and visually displayed using Proksee (Grant et al. 2023) (https://proksee.ca/). The nucleotide sequences of each PCG of *A. castelnaudii* and its related species were aligned using MUSCLE v3.8.31 (Edgar 2004). The best-fit substitution model for each alignment was determined using the ModelFinder model (Kalyaanamoorthy et al. 2017) integrated within IQ-TREE2 (Nguyen et al. 2015). Phylogenetic analysis using Maximum Likelihood (ML) of *A. castelnaudii* and other species of hummingbirds and one outgroup (*Apus apus*) was performed using IQ-TREE2 (Nguyen et al. 2015) based on the sequences from 13 mitochondrial protein-coding genes (PCGs). Individual gene alignments were combined to form a partitioned supermatrix encompassing 11,385 bp. This supermatrix was then employed to construct a ML tree with separate model partitions for each gene. The reliability of the branches within the tree was evaluated using UFBoot2 (Hoang et al. 2018) with 1,000 bootstrap replicates.

## Results

**Figure 2.**
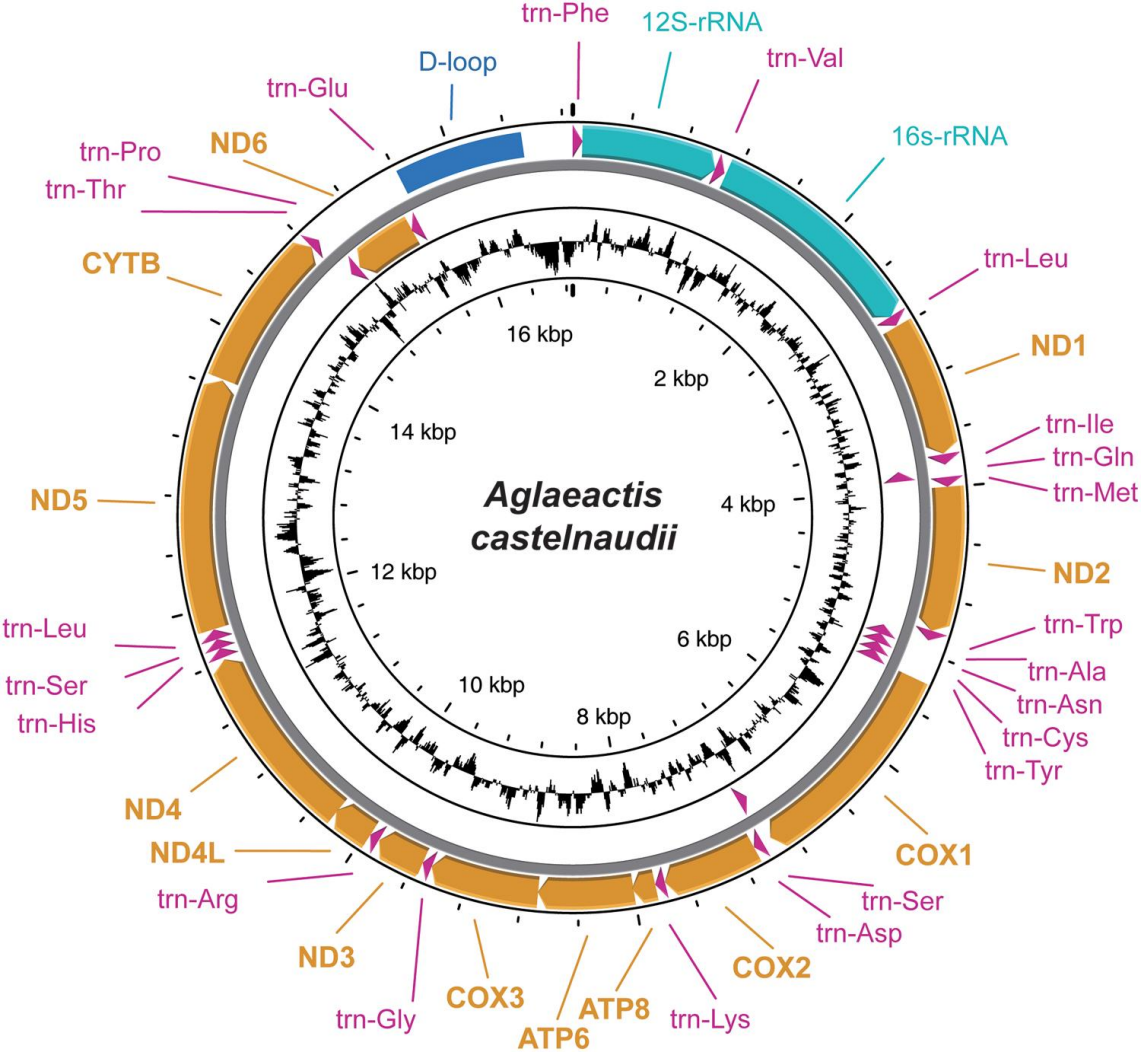
The mitogenome map of *aglaeactis castelnaudii*, drawn by using proksee (Grant et al. 2023) (https://proksee.ca/). GC content was also shown in the figure.

The complete mitogenome of *A. castelnaudii* spans 16,872 bp (NCBI accession number: PP754509). The average read depth coverage for the mitogenome reached 902.9 × (Fig. S1). The mitogenome consists of 13 PCGs, 22 transfer RNA (tRNA) genes, 2 ribosomal RNA (rRNA) genes, and a single control region referred to as the D-loop (Figure 2). This genetic arrangement adheres to the typical structure of avian mitochondrial genes. The nucleotide composition of the genome is as follows: Adenine (A) 30.6%, Thymine (T) 24.0%, Cytosine (C) 31.2%, and Guanine (G) 14.2%, demonstrating a slight bias toward AT content at 54.6%. Notably, among the PCGs, *ND5* is the longest, spanning 1,815 bp, while ATP8 is the shortest at 168 bp. The two rRNA genes (*12S rRNA* and *16S rRNA*) are 974 bp and 1,592 bp in length respectively, positioned between *tRNA^Phe^* and *tRNA^Leu^*, and separated by the *tRNA^Val^* gene. Furthermore, this genomic configuration encompasses 22 transfer RNAs (tRNAs) with lengths ranging from 67 to 80 bp.

## Discussion and conclusion

We assembled and reported the first complete mitogenome of *A. castelnaudii* based on high-throughput genomic data. The genomic orientation, gene arrangement, and nucleotide composition were very similar to other hummingbirds from the genera *Archilochus* (Morgan-Richards et al. 2008), *Chrysolampis* (Souto et al. 2016), and *Amazilia* (Prosdocimi et al. 2016). The A + T content of *A. castelnaudii* was 54.6%, consistent with the slight A + T bias observed in other hummingbird mitogenomes (Souto et al. 2016).

**Figure 3.**
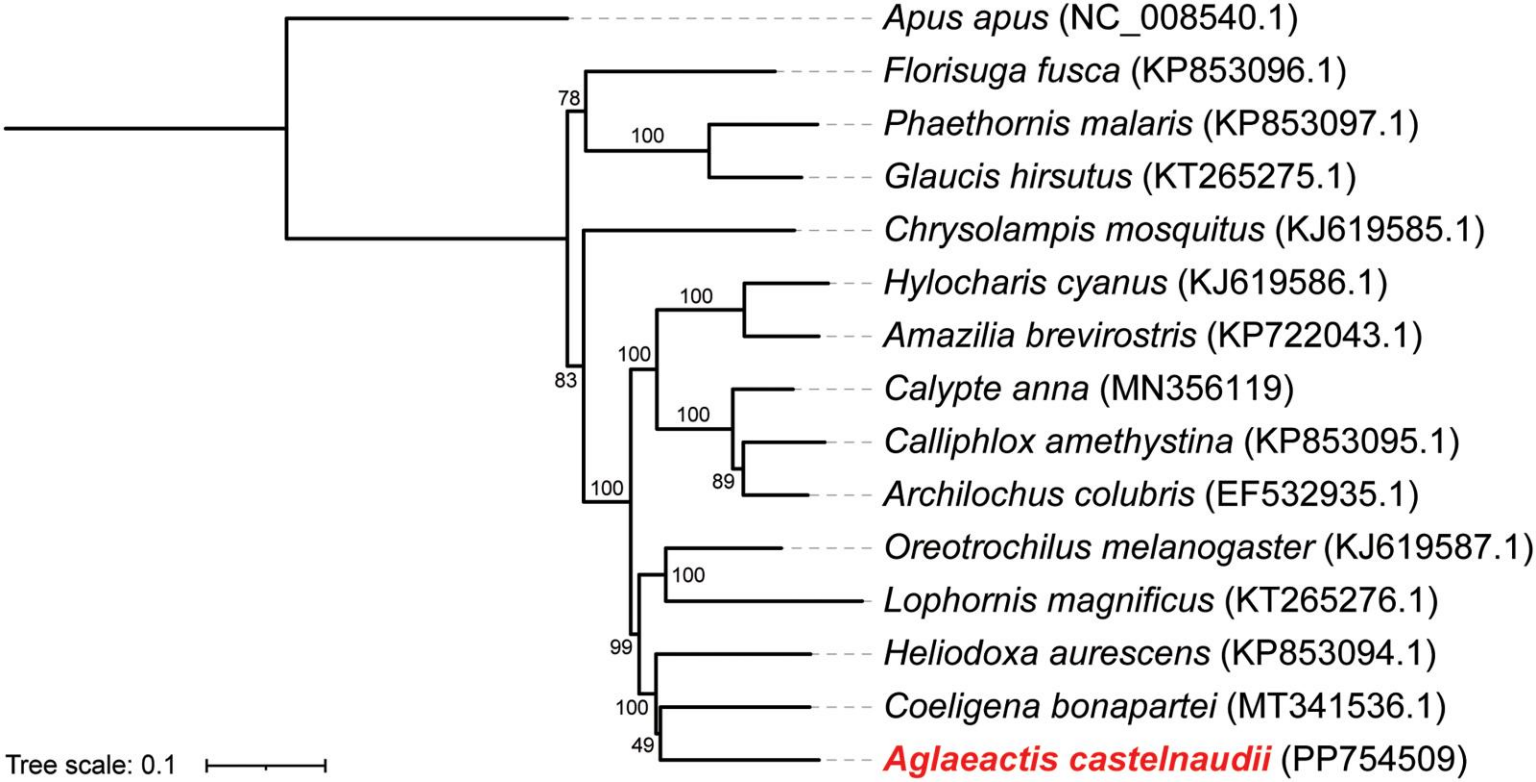
Phylogenetic tree of 14 species belonging to the trochilidae family constructed by using the maximum likelihood (ML) method, utilizing a dataset consisting of 13 PCGs. *Apus apus* from family apodidae was used as outgroup. Bootstrap values were shown alongside to the branches. The following sequences were used to infer the tree: *Amazilia brevirostris* KP722043.1, *archilochus colubris* EF532935.1 (Morgan-Richards et al. 2008), *calliphlox amethystine* KP853095.1, *calypte anna* MN356119 (Feng et al. 2020), *chrysolampis mosquitus* KJ619585.1 (Souto et al. 2016), *coeligena bonapartei* MT341536.1 (Palacios et al. 2023), *florisuga fusca* KP853096.1, *glaucis hirsutus* KT265275.1, *heliodoxa aurescens* KP853094.1, *hylocharis cyanus* KJ619586.1, *lophornis magnificus* KT265276.1, *oreotrochilus melanogaster* KJ619587.1, *phaethornis malaris* KP853097.1, *Apus apus* NC_008540.1 (Slack et al. 2007), and *aglaeactis castelnaudii* PP754509.

The phylogenetic tree supports a closer relationship between *A. castelnaudii* and *Coeligena bonapartei* compared to other species within the Trochilidae family investigated in this study (Figure 3). This contrasts with earlier phylogenetic analyses relying on four nuclear and two mitochondrial genes, which positioned *C. bonapartei* in a closer relationship with *Heliodoxa aurescens*, rather than with *A. castelnaudii* (McGuire et al. 2014). However, the respective topologies in both McGuire et al. (2014) and our study were both poorly supported, and thus require further research. Fundamentally, our study provides a valuable genetic resource for future investigations into the evolutionary trajectory and phylogenetic position of this distinctive hummingbird species exclusively found in the Andes.

## Supplementary Material

Fig S1.tif

## Data Availability

The genomic data used for assembling the mitochondrial genome of *A. castelnaudii* are openly available in SRA of NCBI (https://www.ncbi.nlm.nih.gov/sra/) under the accession number SRR19461672. The associated BioProject and BioSample numbers are PRJNA844086 and SAMN28788517, respectively. Mitogenome of *A. castelnaudii* can be accessed *via* accession number PP754509 in GenBank of NCBI at https://www.ncbi.nlm.nih.gov/nuccore/PP754509.
