# Supplementary figures and images for: The complete mitochondrial genome of aglaeactis castelnaudii (bourcier & mulsant, 1848) (apodiformes: trochilidae: aglaeactis) and phylogenetic analysis
Source: Mitochondrial DNA B Resour. 2024 Sep 4;9(9):1181–4. doi: 10.1080/23802359.2024.2397980 (PMC11376301; doi:10.1080/23802359.2024.2397980)

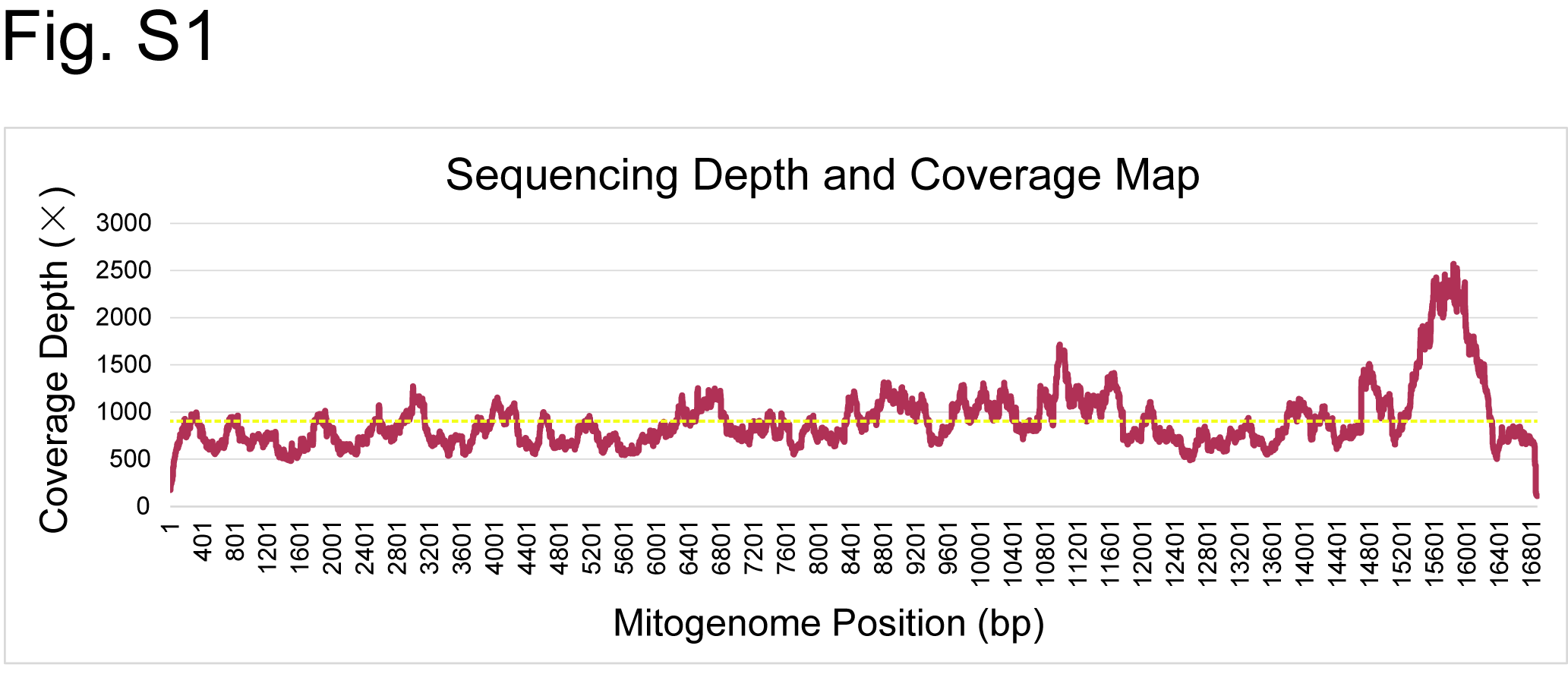

Supplement: Fig S1.tif [file TMDN_A_2397980_SM1884.tif]
